# Supplementary figures and images for: Effect of cotton residues incorporation on soil properties, organic nitrogen fractions, and nitrogen-mineralizing enzyme activity under long-term continuous cotton cropping
Source: PeerJ. 2021 Apr 7;9:e11053. doi: 10.7717/peerj.11053 (PMC8035904; doi:10.7717/peerj.11053)

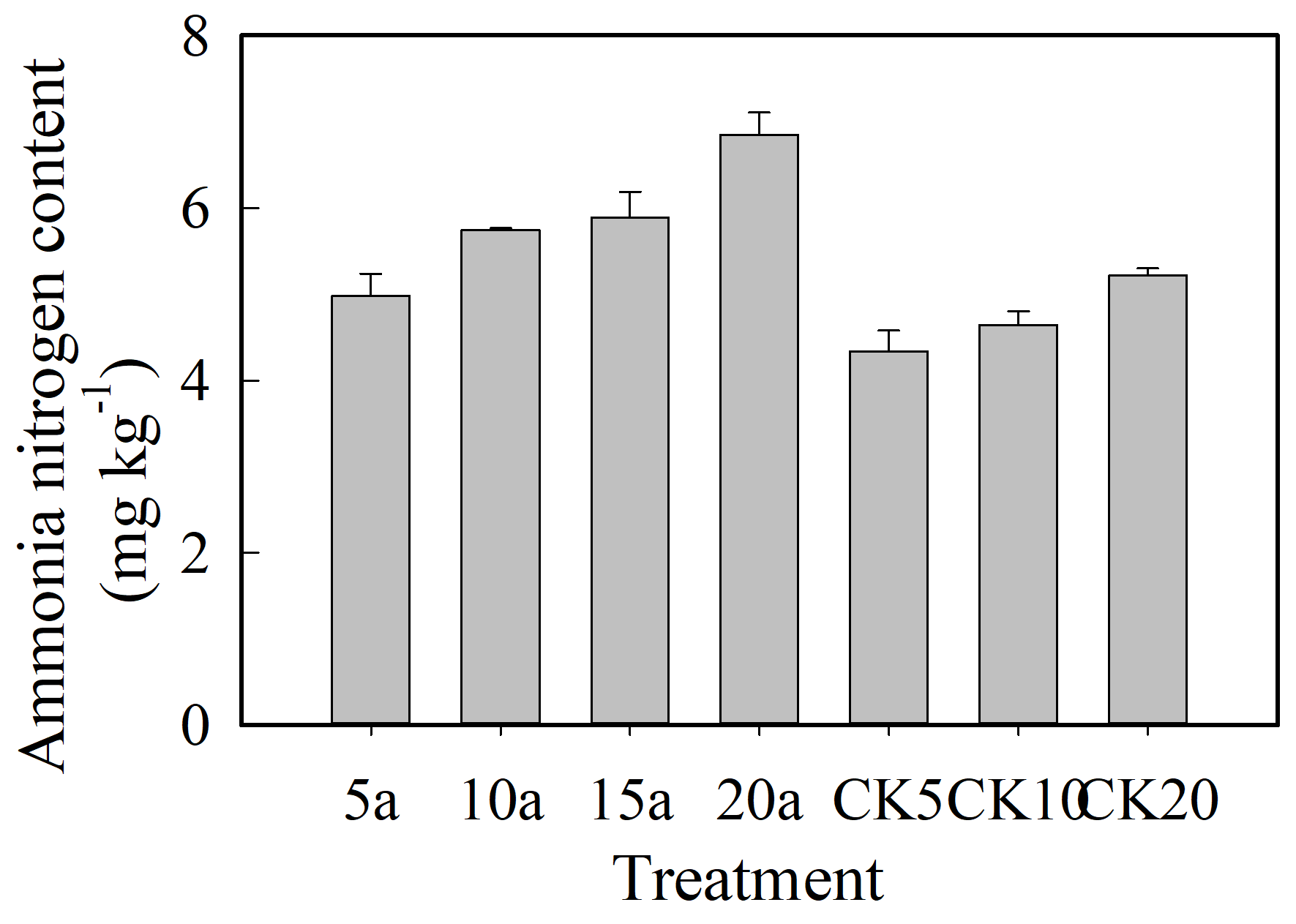

Supplement: Figure S1 [file peerj-09-11053-s002.png]
